# Adhesion of Hydrogels to Fluoropolymers by Electrophoretic Fluoropolymer Coating

**DOI:** 10.1002/marc.70414

**Published:** 2026-08-28

**Authors:** Yoshiaki Kobayashi, Tatsumi Shigenari, Syuuhei Komatsu, Akihiko Kikuchi, Taka‐Aki Asoh

**Affiliations:** ^1^ Department of Materials Science and Technology Tokyo University of Science Katsushika‐ku Tokyo Japan; ^2^ Faculty of Pharmacy and Pharmaceutical Sciences Josai University Sakado Saitama Japan; ^3^ Department of Medical and Robotic Engineering Design Tokyo University of Science Katsushika‐ku Tokyo Japan

**Keywords:** adhesion, coating, electrophoresis, fluoropolymers, hydrogels

## Abstract

Although hydrogels are widely used in bio‐integrated devices and soft systems, their robust adhesion to polymers with low surface energies remains a fundamental challenge. Fluoropolymers such as polytetrafluoroethylene (PTFE) are notoriously difficult to adhere to because of their chemical inertness and non‐wetting surfaces. In this study, we demonstrate that water‐stable adhesion between hydrogels and fluoropolymers can be achieved by engineering interfacial dipole–dipole interactions. In particular, a fluoropolymer coating was deposited on hydrogel surfaces via the electrophoretic deposition of an anionic fluoro‐copolymer, resulting in a polyion‐complexed interfacial layer. The coated hydrogels exhibited stable adhesion to PTFE, even in water. Notably, adhesion to PTFE displayed an on/off transition with slight changes in copolymer composition, while adhesion to other substrates remained unaffected. This unique composition sensitivity revealed that adhesion to PTFE required exceeding the critical surface density of fluorinated dipoles, which distinguishes it from conventional wetting‐driven adhesion mechanisms. Consequently, this study highlights dipole–dipole interactions as a decisive design principle for bonding hydrogels to chemically inert fluoropolymers.

## Graphical abstract

Both fluoropolymers and hydrogels are generally regarded as difficult‐to‐bond materials; however, adhesion between them was achieved by coating the hydrogel with a fluorinated copolymer.

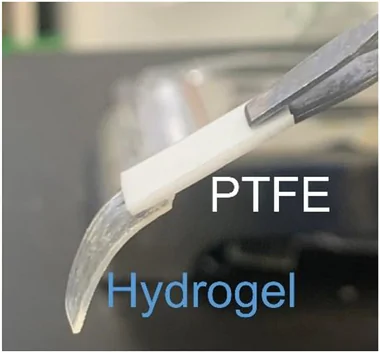

## 1 Introduction

Hydrogels exhibit intrinsic softness, biocompatibility, and permeability, rendering them highly attractive for the development of medical devices, energy harvesters, and soft actuators [1, 2, 3, 4, 5, 6]. In particular, hydrogels are often integrated with solid substrates to improve the structural robustness of the product [7, 8]. Developing adhesion strategies between hydrogels and solid substrates remains essential; however, the abundant number of water molecules within hydrogels often hinders their adhesion. At hydrogel surfaces, interfacial water screens key intermolecular interactions such as hydrogen bonding, van der Waals interactions, and electrostatic interactions while dynamically exchanging at the interface, destabilizing any interactions formed with the polymer network of the hydrogel. Although considerable efforts have been made to develop methods for hydrogel–metal, hydrogel–ceramic, and hydrogel–plastic adhesion [9], their effectiveness is often limited when the counter surface comprises polymer materials with low surface energies [10].

The integration of hydrogels with low‐surface‐energy polymer materials, such as fluoropolymers, has attracted increasing interest for emerging applications, including implantable devices, energy devices, and soft robotics, where chemical stability and mechanical softness are simultaneously required [11, 12, 13, 14, 15, 16]. Such hydrogel–fluoropolymer hybrid devices require material designs that combine the biocompatible and permeable interfaces of hydrogels with the chemical stability and antifouling surfaces of fluoropolymers. Because hydrogels function in their hydrated state, these devices are often required to operate in water‐rich environments; however, interfacial water can weaken adhesion and cause delamination between the hydrogel and fluoropolymer components. This challenge is particularly pronounced for fluoropolymers, which are intrinsically difficult to bond to because of their chemical inertness and non‐wetting properties [17]. Therefore, strategies that modify hydrogel surfaces to enable stable adhesion to fluoropolymers are highly desirable. Recently, Akimoto et al. [18] introduced a nanophase‐separated structure on hydrogel surfaces by grafting a fluoropolymer via surface‐initiated atom transfer radical polymerization, which imparted water repellency to the hydrogel surface.

Limited studies have reported on the adhesion between hydrogels and fluoropolymers. One such study demonstrated that incorporating catechol groups into hydrogels can promote their adhesion to fluoropolymers through hydrophobic interactions [10, 19, 20, 21]. Although catechol groups can adhere to a wide range of substrates, their universality limits the design of substrate‐specific interactions. Moreover, for fluoropolymers with chemical inertness and low surface energy, the lack of tailored interfacial interactions renders it difficult to rationally control and optimize adhesion performance.

In contrast to these approaches, dipole–dipole interactions may offer a promising route for the adhesion of fluoropolymers [22, 23]. Owing to the high electronegativity of fluorine, C─F bonds exhibit a large dipole moment and engage in electrostatic interactions, such as dipole–dipole and ion–dipole interactions [24]. Wang et al. [23] demonstrated that dipole–dipole interactions could be employed to fabricate elastomers capable of self‐healing, even in water. Inspired by these findings, we propose a strategy for depositing a fluoropolymer coating onto hydrogel surfaces via an electrophoretic approach, enabling adhesion to fluoropolymers through interfacial dipole–dipole interactions (Figure 1). Previously, we reported a simple method for forming a polyion complex on hydrogel surfaces using electrophoresis, enabling the formation of wrinkled structures [25, 26, 27]. Under an applied electric field, cationic and anionic polymers migrate toward oppositely charged electrodes and subsequently diffuse across the interface, where they spontaneously form robust polyion complexes [28, 29, 30, 31]. This method offers significant advantages, such as being rapid, washing‐free, and compatible with soft and delicate materials. In this study, an anionic fluoro‐copolymer was electrophoretically driven toward the surface of a cationic hydrogel, resulting in the formation of a polyion complex that functioned as an adhesive layer. This approach resulted in the stable adhesion of hydrogels to polytetrafluoroethylene (PTFE), even in water. Furthermore, the adhesion behavior could be tuned by controlling the copolymer composition of the fluoropolymer. This strategy provides a simple and versatile route for integrating hydrogels with chemically inert fluoropolymer substrates without requiring surface modifications. In addition to demonstrating adhesion to PTFE, we systematically examine how electrophoretic deposition conditions, hydrogel composition, and fluoropolymer composition govern adhesion behavior. Through these investigations, we clarify the key interfacial factors required for hydrogel–fluoropolymer adhesion.

**FIGURE 1:**
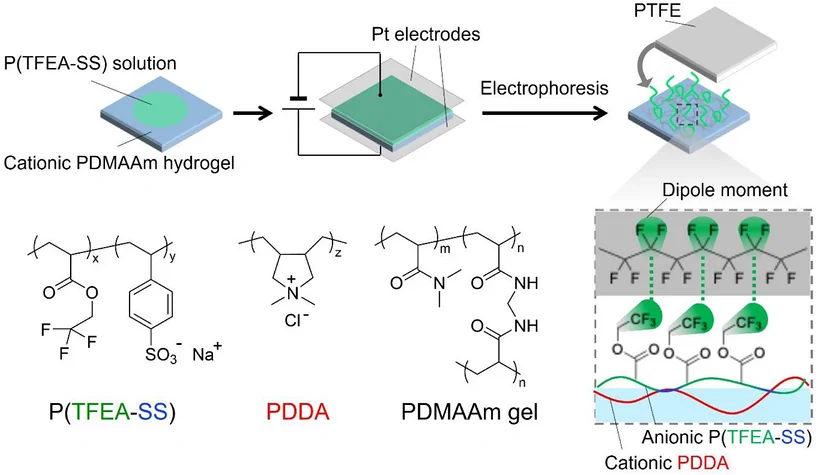
Schematic illustration of adhesion between the hydrogel and PTFE substrate via dipole–dipole interactions among the anionic fluoro‐copolymer on the hydrogel surface and PTFE.

## 2 Results and Discussion

A fluoropolymer coating was deposited onto the hydrogel surface via electrophoresis, as shown in Figure 1. Briefly, a methanol solution of poly((2,2,2‐trifluoroethyl acrylate)‐*co*‐(sodium *p*‐styrenesulfonate)) [P(TFEA‐SS)] was dropped onto the surface of poly(*N,N*‐dimethyl acrylamide) (PDMAAm) hydrogels containing poly(diallyldimethylammonium chloride) (PDDA), which were sandwiched between two electrodes. An electric field was applied to the cationic PDMAAm hydrogel and methanol solution sides as the anode and cathode, respectively. Water contact angles were measured to assess the changes in surface wettability associated with the formation of polyion complexes. The bare cationic hydrogel surface was found to be hydrophilic, whereas the P(TFEA‐SS)‐coated hydrogel surface showed hydrophobicity (Figure 2a). The water contact angle increased monotonically with increasing electric field magnitude, followed by saturation at 8 V mm^−1^ (Figure 2b). The increase in water contact angle reflected an increase in the surface coverage and density of the fluorinated segments resulting from the formation of the P(TFEA‐SS)/PDDA polyion complex. After applying an electric field, P(TFEA‐SS) and PDDA migrated toward the anode and cathode, respectively (Figure 2c). These polyions diffused into the opposing material, resulting in the formation of a P(TFEA‐SS)/PDDA complex at the interface. Therefore, P(TFEA‐SS) was successfully coated on the cationic hydrogel surface, and the surface density of the fluoropolymer‐based polyion complex could be quantitatively controlled by applying an electric field.

**FIGURE 2:**
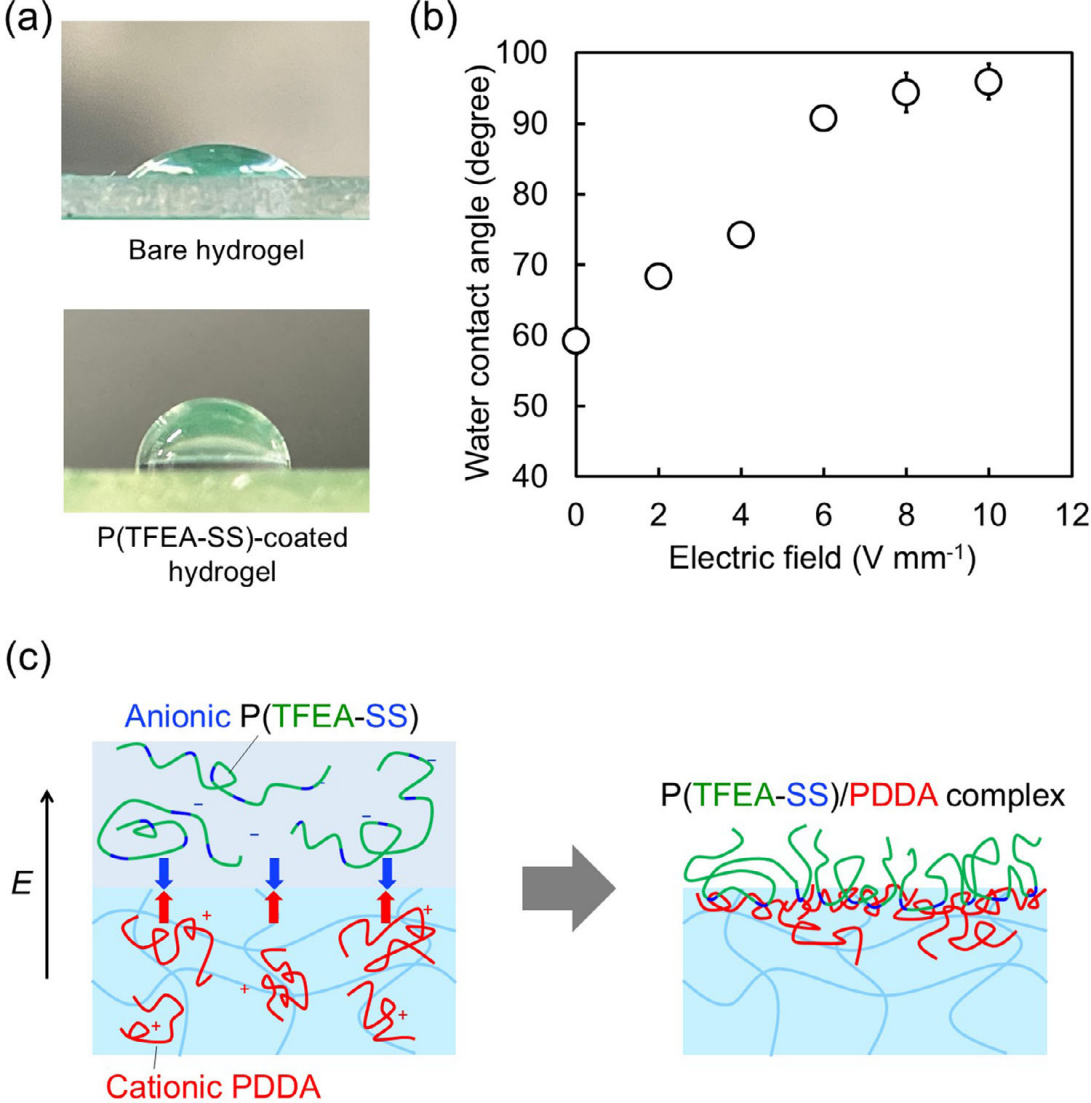
(a) Water droplets on uncoated (top) and P(TFEA‐SS)‐coated (bottom) hydrogels. (b) Water contact angle as a function of the electric field applied during the formation of the P(TFEA‐SS) coating. (c) Schematic illustration of the electrophoretic formation of a P(TFEA‐SS)/PDDA polyion complex on the hydrogel surface, resulting in the enrichment of fluorinated dipoles at the interface.

Adhesion was observed only when excess surface water was removed prior to contact with the PTFE substrate, whereas no measurable adhesion was obtained when bonding was attempted entirely under water. To compare adhesion under dry and underwater adhesion conditions, the coated hydrogel and PTFE substrate were brought into contact entirely underwater. In this case, no measurable adhesion was observed. In contrast, strong adhesion was obtained when the interface was formed under ambient conditions, and the adhered samples remained adhesion after subsequent immersion in water. This behavior suggests that the formation of the adhesive interface requires direct interfacial contact between the fluorinated moieties and the PTFE surface. Under aqueous conditions, the sulfonate groups of P(TFEA‐SS) are likely hydrated and exposed at the interface, forming a hydration layer that hinders intimate contact with the PTFE surface. Once the adhesive interface is established under ambient conditions, however, the resulting interfacial interactions are sufficiently stable to resist debonding upon subsequent water immersion.

To establish hydrogel–PTFE adhesion as a function of interfacial coating formation, we systematically investigated the effects of electrophoresis conditions, polycation concentration, and hydrogel crosslinking density on the adhesion behavior. Figure 3 summarizes the adhesion maps obtained under these conditions and demonstrates how interfacial polyion complex formation governs hydrogel–PTFE adhesion. A simple adhesion test consisting of water agitation followed by peeling with tweezers was conducted to evaluate the adhesion properties of the P(TFEA‐SS)‐coated hydrogels (Figure 3a).

**FIGURE 3:**
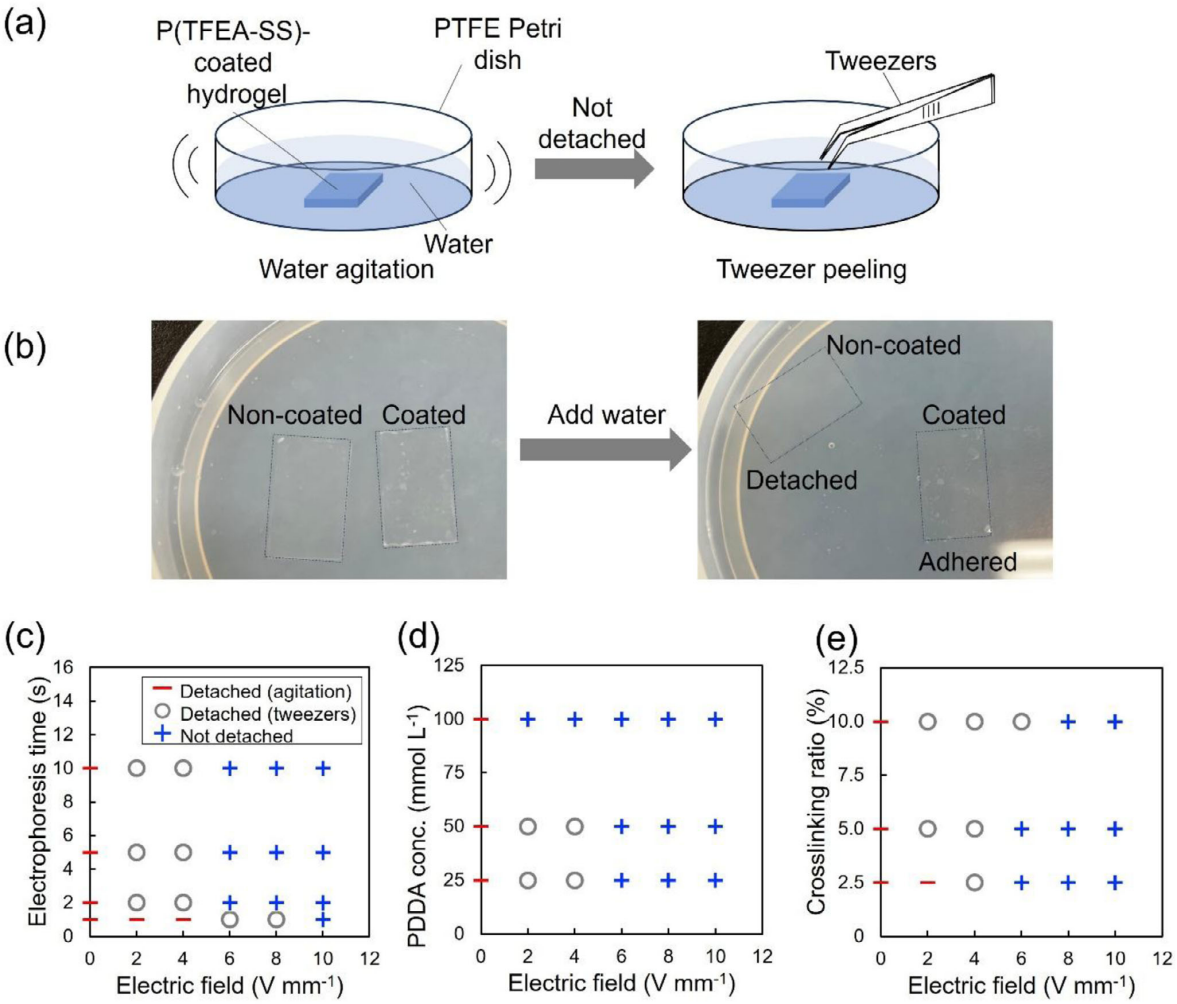
(a) Illustration of a simple adhesion test. (b) Representative photographs show that both samples adhere in air, whereas the non‐coated hydrogel detaches upon immersion in water. In contrast, the fluorine‐coated hydrogel remains attached, demonstrating enhanced underwater adhesion stability. Adhesion maps of (c) electrophoresis time, (d) PDDA concentration, and (e) hydrogel crosslinking density, which regulated adhesion by controlling the effective interfacial polyion complex density. The symbols indicate the adhesion state: detached by water agitation (red minus sign), detached by tweezer peeling (gray circle), and not detached (blue plus sign).

The P(TFEA‐SS)‐coated hydrogel prepared under an electric field of 10 V mm^−^^1^ for 10 s remained firmly adhered to the PTFE surface after water agitation (Figure 3b). Subsequent tweezer peeling test revealed that cohesive fracture occurred within the hydrogel rather than interfacial failure, leaving part of the gel attached to the PTFE substrate. In contrast, the bare cationic hydrogel detached readily from the PTFE surface during water agitation, indicating that surface modification by the fluoropolymer coating was essential for adhesion.

The adhesion behavior showed a strong dependence on both the magnitude of the applied electric field and electrophoresis time (Figure 3c). For example, increasing the electric field strength and electrophoresis duration promoted adhesion to the PTFE surface. At 10 V mm^−^^1^, stable adhesion was achieved even with an electrophoresis time as short as 1 s, because no detachment was observed upon tweezer peeling. Thus, stable adhesion emerged once the density of the interfacial polyion complex exceeded a threshold value. Moreover, the observed adhesion is consistent with the contribution of dipole‐mediated interactions between the fluorinated segments in P(TFEA‐SS) and the PTFE surface.

To investigate the factors governing hydrogel adhesion to PTFE, we examined the dependence of adhesion behavior on the intrinsic hydrogel parameters. We first investigated the role of the polycation concentration. As shown in Figure 3d, at a fixed electrophoresis time of 10 s, the P(TFEA‐SS)‐coated hydrogels prepared with PDDA concentrations of 25 and 50 mmol L^−1^ exhibited adhesion only at electric field magnitudes ≥6 V mm^−1^, whereas a higher PDDA concentration of 100 mmol L^−1^ resulted in observable adhesion, even at 2 V mm^−1^. Therefore, a higher PDDA concentration promoted the formation of an interfacial P(TFEA‐SS)/PDDA complex, enabling adhesion to PTFE even at lower electric field magnitudes.

Next, we examined how the crosslinking density of the hydrogel network affected the adhesion behavior. As shown in Figure 3e, hydrogels prepared with crosslinking ratios of 2.5% and 5.0% exhibited nearly identical adhesion behaviors, with adhesion emerging only at an electric field magnitude of ≥6 V mm^−1^. In contrast, hydrogels prepared with a higher crosslinking ratio of 10% only exhibited adhesion at 8 V mm^−1^. These results imply that increasing the crosslinking density restricted the mobility of the polycations within the hydrogel network, thereby increasing the magnitude of the electric field required to reach the polyion complex density necessary for adhesion to PTFE. To verify this hypothesis, we determined swelling ratios and Young's moduli of bare cationic hydrogels. As the crosslinking ratios increased from 2.5% to 5.0% and then to 10%, swelling ratios decreased from 14.0 to 10.4 and then to 7.4, respectively. The corresponding water contents were calculated to be 92.9%, 90.4%, and 86.5%, respectively. Furthermore, Young's moduli increased from 99 ± 12 kPa to 266 ± 112 kPa and then to 316 ± 154 kPa with crosslinking ratios of 2.5%, 5.0%, and 10%, respectively. These results indicate that higher crosslinking ratios reduced the water retention capacity and stiffened the polymer network in hydrogels. Thus, the reduced polymer mobility at higher crosslinking densities limited polycation migration and increased the magnitude of the electric field required for adhesion. Although the parameters shown in Figure 3 vary independently, they all regulated adhesion by controlling the effective density of the interfacial polyion complex.

After identifying the coating parameters required for hydrogel–PTFE adhesion (Figure 3), we further investigated the substrate dependence of adhesion to distinguish general adhesive effects from PTFE‐specific interfacial interactions. The adhesion behavior of the P(TFEA‐SS)‐coated hydrogels was further evaluated on various substrates, including hydrophobic polymers (polypropylene [PP] and polyethylene terephthalate [PET]), hydrophilic materials (glass and aluminum), and PTFE (Figure 4). The coated hydrogels exhibited comparable adhesive strengths toward PP, PET, glass, and aluminum irrespective of the copolymer composition. This composition‐insensitive adhesion was attributed to the low glass transition temperature of P(TFEA‐SS), which enabled intimate interfacial contact and promoted generic adhesion through viscoelastic dissipation and interfacial wetting even on substrates with contrasting surface chemistries. In sharp contrast, adhesion to PTFE displayed a pronounced dependence on the copolymer composition. Although no measurable adhesion was observed for P(TFEA‐SS) with a composition of 95:5, a slight increase in the fluorinated segment fraction to 97:3 resulted in stable adhesion to PTFE, with an adhesive strength of 7.4 ± 1.2 kPa. Water contact angle measurements revealed a significant increase from 77.9° to 95.9° with this compositional change, indicating an enhanced surface enrichment of the fluorinated moieties. Notably, such a drastic on/off transition in adhesion with a minimal compositional change was observed only for PTFE and not for the other substrates. The sharp transition in adhesion observed between the 95:5 and 97:3 compositions suggests the existence of a critical balance between the fluorinated moieties and the ionic SS units. At relatively high SS contents, hydrated sulfonate groups may be enriched at the interface, which may hinder intimate contact between fluorinated segments and the PTFE surface. Although such copolymers can still exhibit adhesion toward other hydrophobic substrates through nonspecific viscoelastic and wetting effects, the excessive ionic character appears to suppress the specific interactions required for PTFE adhesion. As the SS content decreases, the surface density of fluorinated moieties increases. Once a critical fluorinated moiety density is reached, the fluorinated segments become sufficiently exposed at the interface to promote interactions with the PTFE surface, resulting in the abrupt emergence of adhesion. This unique sensitivity indicates that conventional wetting‐driven or viscoelastic adhesion mechanisms that govern adhesion to PP, PET, and inorganic substrates were insufficient to sustain adhesion to PTFE. Instead, adhesion to PTFE appeared to require a critical surface density of fluorinated moieties, suggesting the involvement of specific interactions associated with fluorinated groups. Therefore, the adhesion behavior toward PTFE likely involves interfacial interactions distinct from those responsible for adhesion to the other substrates examined in this study.

**FIGURE 4:**
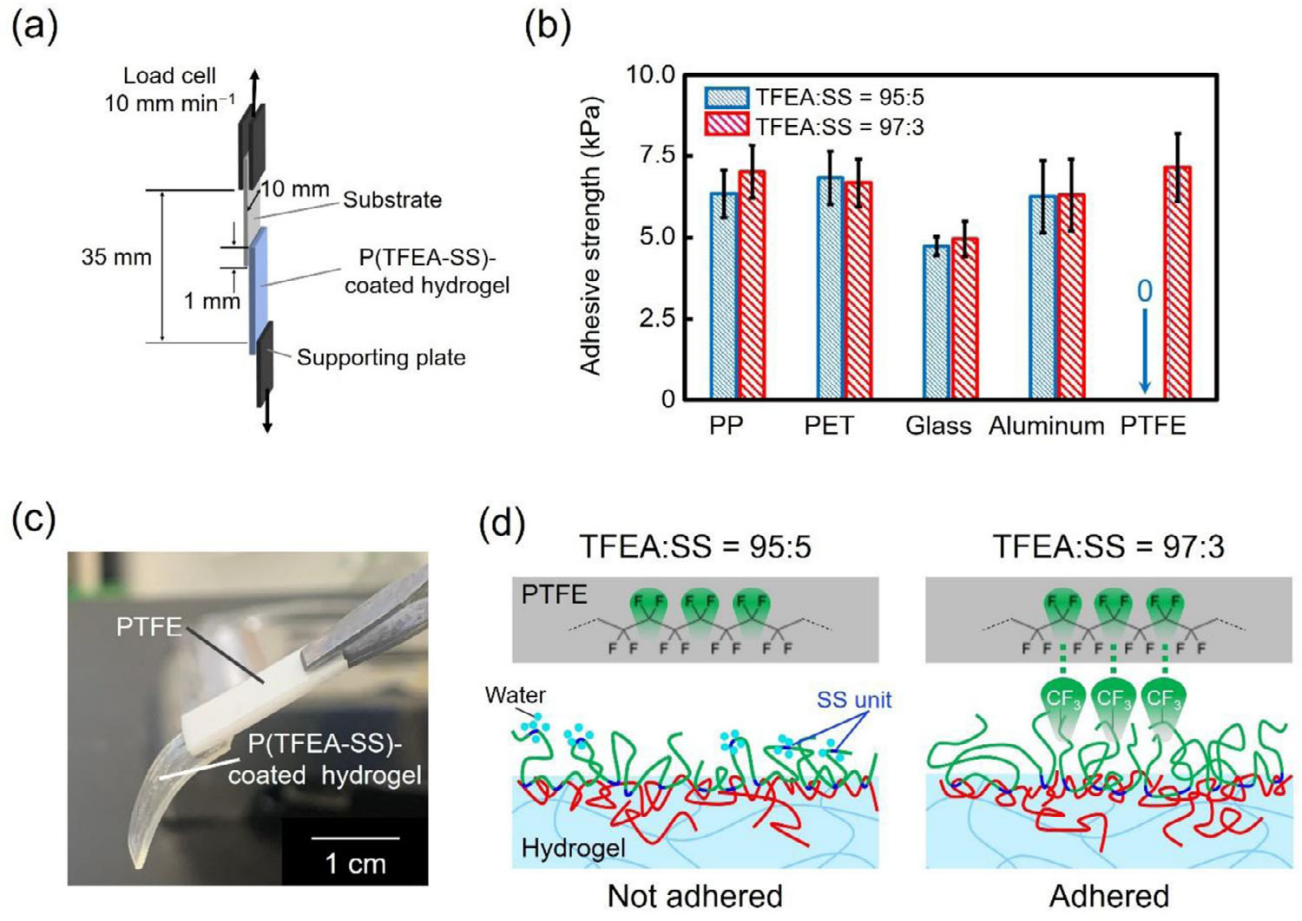
(a) Experimental procedure for the lap shear tests of bonded P(TFEA‐SS)‐coated hydrogels on various substrates. (b) Comparison of adhesive strength between the composition ratios of P(TFEA‐SS) on different types of substrates. (c) Image of the P(TFEA‐SS)‐coated hydrogel adhered to a PTFE substrate. (d) Schematic illustration of fluoropolymer coatings with different copolymer compositions. Variations in fluorinated segment content alter the surface properties of the coating and influence the resistance to water intrusion at the hydrogel/substrate interface.

To evaluate the stability of the hydrogel–PTFE interface under aqueous conditions, adhered hydrogels were immersed in water for one week. No observable interfacial delamination was detected during this period, indicating that the formed interface remained stable under prolonged water exposure. In contrast, strongly adhered specimens typically failed through cohesive fracture of the hydrogel rather than interfacial debonding during adhesion testing. Therefore, repeated loading tests on the same specimen were not feasible because the hydrogel itself fractured before interfacial failure occurred. Further studies on longer‐term aging and durability under aqueous environments are subjects of future investigation.

The present electrophoretic coating approach enabled the precise control of the surface dipole density via copolymer composition, thereby selectively activating dipole–dipole interactions at the hydrogel–PTFE interface. These results are consistent with the hypothesis that dipole‐mediated interactions contribute to hydrogel–PTFE adhesion. However, direct spectroscopic verification of the interfacial interactions remains an important subject for future study.

## 3 Conclusions

We successfully formed a fluoropolymer coating on hydrogel surfaces via the electrophoretic deposition of a polyion complex comprising anionic fluoropolymers and cationic polymers. The fluoropolymer coating transformed the hydrogel surface from hydrophilic to hydrophobic, and the covering density of the coating was controlled by tuning the applied electric field. The fluoropolymer‐coated hydrogels exhibited stable adhesion to PTFE in water. The adhesion behavior was influenced by the interfacial polyion complex density, with adhesion enhanced by a higher electric field strength, longer electrophoresis time, increased polycation concentration, and lower crosslinking density of the hydrogel network. In addition to PTFE, the fluoropolymer‐coated hydrogels exhibited adhesive strengths comparable to those of various substrates, including PP, PET, glass, and aluminum, demonstrating the general applicability of this approach. Their adhesion to PTFE was highly sensitive to the copolymer composition. For example, a slight increase in the hydrophilic component suppressed adhesion, highlighting the importance of specific interactions associated with fluorinated moieties, which may include dipole‐mediated interactions.

Overall, this study established electrophoretic interfacial complexation as a versatile platform for tailoring hydrogel surface properties and enabling adhesion to a wide range of substrates, including chemically inert fluoropolymers, under mild and aqueous conditions. This strategy offers new opportunities for integrating hydrogels with diverse materials in applications such as bio‐integrated devices and soft robotics.

## 4 Experimental Section

### 4.1 Materials

*N*,*N*‐dimethyl acrylamide (DMAAm) was purchased from FUJIFILM Wako Pure Chemical Corporation and distilled prior to use. 2,2,2‐Trifluoroethyl acrylate (TFEA), 2‐hydroxy‐4‐(2‐hydroxyethoxy)‐2‐methylpropiophenone (Irgacure 2959), and dimethyl sulfoxide‐d6 were purchased from Tokyo Chemical Industry Co., Ltd. and used as received without further purification. Sodium p‐styrene sulfonate (SS), N,N‐methylenebis(acrylamide) (MBAAm), 2,2’‐azobis(isobutyronitrile) (AIBN), and dimethyl sulfoxide (DMSO) were purchased from FUJIFILM Wako Pure Chemical Corporation and used as received. Methanol was purchased from KANTO Chemical Co., Inc., while poly(diallyldimethylammonium chloride) (PDDA) was purchased from Sigma–Aldrich as an aqueous solution and used after lyophilization.

### 4.2 Preparation and Characterization of the Cationic PDMAAm Hydrogels

Cationic PDMAAm hydrogels with semi‐interpenetrated network structures were prepared via the free‐radical polymerization of DMAAm with MBAAm in the presence of PDDA using Irgacure 2959 as the photopolymerization initiator. All the reagents were dissolved in water and degassed under vacuum. The degassed solutions were poured into a glass and silicone elastomer spacer mold (90 mm × 90 mm × 1 mm). UV light (20 mW cm^−2^ at 365 nm; HOLOLIGHT KAKU, Pi PHOTONICS, Inc.) was applied for 60 min, and the mold was flipped every 15 min. The prepared hydrogels were cut into 20 × 10 mm pieces and subjected to electrophoresis. The Young's modulus of each gel was measured using a tensile tester (Force Tester MCT‐2150 W, A&D Company) under ambient atmosphere conditions at a speed of 10 mm min^−^^1^. For each hydrogel, three measurements were performed, and the results are reported as the mean value. The swelling ratios were determined from the weights of the swollen and dried hydrogels.

### 4.3 Preparation of Poly(2,2,2‐Trifluoroethyl Acrylate‐Co‐Sodium p‐Styrenesulfonate) [P(TFEA‐SS)]

The copolymerization of TFEA and SS was performed in DMSO using 1 mol% AIBN as an initiator under a nitrogen atmosphere at 70 °C for 24 h. The product was subsequently purified via dialysis using a cellulose membrane with a molecular weight cutoff of 3500. Dialysis was conducted in methanol for three days followed by water for an additional three days, and the external medium was replaced every 24 h. The dialyzed solution was concentrated using a rotary vacuum evaporator (N‐1300, Tokyo Rikakikai Co., Ltd.), and the polymer was obtained by lyophilization (FDU‐1100, Tokyo Rikakikai Co., Ltd.). The chemical structure and composition of the P(TFEA‐SS) copolymer were determined using proton nuclear magnetic resonance (1H NMR, Bruker Avance Neo 400, Bruker) spectroscopy.

### 4.4 P(TFEA‐SS) Coating on the Cationic PDMAAm Hydrogel

A 1 wt.% methanol solution of P(TFEA‐SS) was dropped onto the surface of the cationic PDMAAm hydrogel. The system was sandwiched between two Pt electrodes, and an electric field was applied. The cationic PDMAAm hydrogel side served as the anode while the methanol solution side served as the cathode. The contact angle was measured three times, and the results are reported as the mean value.

### 4.5 Simple Adhesion Test

The P(TFEA‐SS)‐coated hydrogels were gently blotted with a paper wipe to remove excess surface water and then placed on a PTFE Petri dish with the coated surface facing the PTFE side. The dish was then filled with distilled water and gently shaken for 10 s, followed by an examination of the adhesion state. If the hydrogel remained attached to the PTFE surface, it was removed using tweezers. The results were classified into three categories: (i) detached by water agitation, (ii) not detached by water agitation, but detached by tweezer peeling, and (iii) not detached by tweezer peeling.

### 4.6 Measurement of Adhesion Strength

The adhesion strength was measured via lap shear tests using a tensile tester. The P(TFEA‐SS)‐coated surfaces of the hydrogels were attached to PP, PET, glass, aluminum, and PTFE substrates with a controlled contact area of 10 mm^2^, and lap shear measurements were performed under identical test conditions. Three measurements were performed, and the results are reported as the mean value.

## Conflicts of Interest

The authors declare no conflicts of interest.

## Data Availability

The data that support the findings of this study are available on request from the corresponding author. The data are not publicly available due to privacy or ethical restrictions.

## References

[1] H. M. Appel , J. S. Tibbitt , B. T. Langer , D. G. Anderson , and D. J. Mooney , “Translational Applications of Hydrogels,” Chemical Reviews 121 (2021): 11385. 10.1021/acs.chemrev.0c01177 PMC8461619 33938724

[2] W. Xu , L.‐B. Huang , M.‐C. Wong , L. Chen , G. X. Bai , and J. Hao , “Environmentally Friendly Hydrogel‐Based Triboelectric Nanogenerators for Versatile Energy Harvesting and Self‐Powered Sensors,” Advanced Energy Materials 7 (2017): 1601529, 10.1002/aenm.201601529.

[3] X. Pu , M. Liu , X. Chen , et al., “Ultrastretchable, Transparent Triboelectric Nanogenerator as Electronic Skin for Biomechanical Energy Harvesting and Tactile Sensing,” Science Advances 3 (2017): 1700015. 10.1126/sciadv.1700015 PMC5451198 28580425

[4] Y. Osada , H. Okuzaki , and H. Hori , “A Polymer Gel with Electrically Driven Motility,” Nature 355 (1992): 242–244, 10.1038/355242a0.

[5] Z. Hu , X. Zhang , and Y. Li , “Synthesis and Application of Modulated Polymer Gels,” Science 269 (1995): 525–527, 10.1126/science.269.5223.525. 17842364

[6] T. Asoh , M. Matsusaki , T. Kaneko , and M. Akashi , “Fabrication of Temperature‐Responsive Bending Hydrogels with a Nanostructured Gradient,” Advanced Materials 20 (2008): 2080–2083, 10.1002/adma.200702727.

[7] H. Cao , L. Duan , Y. Zhang , J. Cao , and K. Zhang , “Current Hydrogel Advances in Physicochemical and Biological Response‐Driven Biomedical Application Diversity,” Signal Transduction and Targeted Therapy 6 (2021): 426, 10.1038/s41392-021-00830-x. PMC8674418 34916490

[8] H. Yuk , T. Zhang , G. A. Parada , X. Liu , and X. Zhao , “Skin‐Inspired Hydrogel–elastomer Hybrids with Robust Interfaces and Functional Microstructures,” Nature Communications 7 (2016): 12028, 10.1038/ncomms12028. PMC4931236 27345380

[9] J. Yang , R. Bai , B. Chen , and Z. Suo , “Hydrogel Adhesion: a Supramolecular Synergy of Chemistry, Topology, and Mechanics,” Advanced Functional Materials 30 (2020): 1901693, 10.1002/adfm.201901693.

[10] X. He , L. Liu , H. Han , W. Shi , W. Yang , and X. Lu , “Bioinspired and Microgel‐Tackified Adhesive Hydrogel with Rapid Self‐Healing and High Stretchability,” Macromolecules 52 (2019): 72–80, 10.1021/acs.macromol.8b01678.

[11] Y. Gao , D. Liu , J. Zhou , et al., “Instant and Secure Fixation of E‐PTFE Medical Implants in Surgery by Designing a Bioadhesive Interface,” Science Advances 11 (2025): adw3845, 10.1126/sciadv.adw3845. PMC12180491 40540558

[12] Z. Li , J. Giarto , J. Zhang , et al., “Anti‐Thrombotic Poly(AAm‐co‐NaAMPS)‐Xanthan Hydrogel‐Expanded Polytetrafluoroethylene (ePTFE) Vascular Grafts with Enhanced Endothelialization and Hemocompatibility Properties,” Biomaterials Advances 154 (2023): 213625. 10.1016/j.bioadv.2023.213625 PMC10841274 37722163

[13] C. Liu , S. Wang , X. Wang , et al., “Hydrovoltaic Energy Harvesting from Moisture Flow Using an Ionic Polymer–hydrogel–carbon Composite,” Energy & Environmental Science 15 (2022): 2489–2498, 10.1039/D2EE00030J.

[14] J. Yu , M. Chen , N. Hu , et al., “Long‐Lasting Hydrogen Evolution and Efficient Dew Harvest Realized via Electrospinning Polyvinylidene Fluoride Membrane on Hybrid Hydrogels,” Small Science 4 (2024): 2400046, 10.1002/smsc.202400046. PMC11935092 40212115

[15] P. Zhang , I. M. Lei , G. Chen , et al., “Integrated 3D Printing of Flexible Electroluminescent Devices and Soft Robots,” Nature Communications 13 (2022): 4775, 10.1038/s41467-022-32126-1. PMC9399151 35999212

[16] X. Fu , G. Wan , H. Guo , et al., “Self‐Healing Actuatable Electroluminescent Fibres,” Nature Communications 15 (2024): 10498, 10.1038/s41467-024-53955-2. PMC11615400 39627213

[17] X. Jin , J. Strueben , L. Heepe , et al., “Joining the Un‐Joinable: Adhesion between Low Surface Energy Polymers Using Tetrapodal ZnO Linkers,” Advanced Materials 24 (2012): 5676–5680, 10.1002/adma.201201780. 22927220

[18] T. Nishimoto , T. Enomoto , C. H. Lin , et al., “Construction of a Nano‐Phase‐Separated Structure on a Hydrogel Surface,” Soft Matter 18 (2022): 722–725, 10.1039/D1SM01659H. 35019926

[19] D. Gan , W. Xing , L. Jiang , et al., “Plant‐Inspired Adhesive and Tough Hydrogel Based on Ag‐Lignin Nanoparticles‐triggered Dynamic Redox Catechol Chemistry,” Nature Communications 10 (2019): 1487, 10.1038/s41467-019-09351-2. PMC6445137 30940814

[20] H. Pang , C. Ma , S. Li , et al., “Tough Thermosensitive Hydrogel with Excellent Adhesion to Low‐Energy Surface Developed via Nanoparticle‐Induced Dynamic Crosslinking,” Applied Surface Science 560 (2021): 149935.

[21] H. Abe , D. Yoshihara , S. Tottori , and M. Nishizawa , “Mussel‐Inspired Thermo‐Switchable Underwater Adhesive Based on a Janus Hydrogel,” NPG Asia Materials 16 (2024): 49, 10.1038/s41427-024-00569-1.

[22] X. Jing , L. Yao , Y. Sheng , H. Lou , Q. Zhang , and S. Zhu , “Record‐High Adhesion on Low‐Surface‐Energy Substrates through Dipole–Dipole Interactions and Frictional Energy Dissipation in Fluorinated Copolymers,” Advanced Functional Materials 35 (2025): 2507232, 10.1002/adfm.202507232.

[23] Y. Cao , H. Wu , S. I. Allec , B. M. Wong , D. Nguyen , and C. Wang , “A Highly Stretchy, Transparent Elastomer with the Capability to Automatically Self‐Heal Underwater,” Advanced Materials 30 (2018): 1804602, 10.1002/adma.201804602. 30368928

[24] D. O'Hagan , “Understanding Organofluorine Chemistry. An Introduction to the C–F Bond,” Chemical Society Reviews 37 (2008): 308–319. 10.1039/b711844a 18197347

[25] M. Kato , T. Asoh , and H. Uyama , “Electrophoretic Fabrication of an Active and Selective Wrinkle Surface on Hydrogels,” Chemical Communications 55 (2019): 4170–4173, 10.1039/C9CC01307E. 30892314

[26] M. Kato , Y. Kashihara , T. Asoh , and H. Uyama , “Geometry Control of Wrinkle Structures Aligned on Hydrogel Surfaces,” Langmuir 36 (2020): 1467–1473, 10.1021/acs.langmuir.9b03967. 31999120

[27] T. Asoh and Y. Kashihara , “Cellulose‐Nanocrystal‐Promoted Underwater Superoleophobic Wrinkles on Hydrogel Surfaces,” Advanced Materials Interfaces 12 (2025): 00555, 10.1002/admi.202500555.

[28] D. Morales , E. Palleau , M. D. Dickey , and O. D. Velev , “Electro‐Actuated Hydrogel Walkers with Dual Responsive Legs,” Soft Matter 10 (2014): 1337–1348, 10.1039/C3SM51921J. 24651405

[29] A. Xin , R. Zhang , K. Yu , and Q. Wang , “Mechanics of Electrophoresis‐Induced Reversible Hydrogel Adhesion,” Journal of the Mechanics and Physics of Solids 125 (2019): 1–21, 10.1016/j.jmps.2018.12.007.

[30] L. K. Borden , A. Gargava , and S. R. Raghavan , “Reversible Electroadhesion of Hydrogels to Animal Tissues for Suture‐Less Repair of Cuts or Tears,” Nature Communications 12 (2021): 4419, 10.1038/s41467-021-24022-x. PMC8292548 34285208

[31] L. K. Borden , M. G. Nader , F. A. Burni , et al., “Switchable Adhesion of Hydrogels to Plant and Animal Tissues,” Advanced Science 12 (2025): 2411942, 10.1002/advs.202411942. PMC11792046 39644503

